# Meteoric Stones

**Published:** 1845-11

**Authors:** 


METEORIC STONES.

If any reader of our Journal should be in possession of a specimen of the above strange visitants of our earth, he will see from the subjoined circular how to make such a disposition of it, as will subserve the cause of science.

"Gentlemen:—Having been appointed by the Association of American Geologists and Naturalists, at their late meeting in this place, to draw up a report on *Meteoric Stones* and *Meteoric Irons*, I take the liberty of addressing you the following inquiries:

"1. Have any meteoric stones fallen in your vicinity of which specimens have been preserved, but of which no accounts have yet been made public in the scientific journals?

"2. Are there any rumors of such falls in your region, but unsupported hitherto by the finding of the stones?

"3. Have any masses of malleable iron been discovered in the soil? When and where? Are any specimens (in their natural state or otherwise), still preserved?

"4. Do any traditions of such masses among blacksmiths, iron-masters, or others, exist in your neighborhood? (Such masses have often been worked up into farming utensils.)

"5. Do you possess meteoric specimens of any kind? If so, from what localities, and what are their respective weights and shapes?

"6. Can you indicate the existence of any such specimens in the hands of others, which are probably unknown to the public?

"For any information on either of the foregoing heads, I shall hold myself deeply obliged; and shall not omit to acknoweledge the same, in the report I may have the honor to submit to the scientific body above referred to.

"Please to address me from May to November, in this city; and the remainder of the year, in Charleston, S. C.

"Very respectfully,
"Your obedient servant,
"CHARLES UPHAM SHEPARD.

"*New Haven, September 17th*, 1845.

"P. S. In order to facilitate researches for the discovery of meteoric stones and irons, Mr. Shepard will all times, cheerfully incur an expense of any sum less than fifty dollars, for specimens from a new locality.

METEORIC COLLECTION OF CHARLES UPHAM SHEPARD, SEPT. 17, 1845.

*Meteoric Stones.*

| | | Date of fall. |
|---|---|---|
| 1. | Barbotan, Dept. du Gers, France, - - - | July 24, 1790. |
| 2. | L'Aigle, Dept. de l'Orne, France, - - - | April 26, 1803. |
| 3. | Weston, Connecticut, U. S. - - - - - | Dec. 14, 1807. |
| 4. | Stannern, Moravia, - - - - - - | May 22, 1808. |
| 5. | Tipperary, Ireland, - - - - - - | Aug. —, 1810. |
| 6. | Chantonnay, Dept. Vendeé, France, - - | Aug. 5, 1812. |
| 7. | Nanjemoy, Maryland, U. S. - - - - | Feb. 10, 1825. |
| 8. | Hondolulu, Owyhee, Sandwich Islands, - | Sept. 15, 1825. |
| 9. | Sumner county, Tennessee, U. S. - - | May 9, 1827. |
| 10. | Richmond, Virginia, U. S. - - - - - | June 4, 1828. |
| 11. | Forsyth, Georgia, U. S. - - - - - | May 8, 1829. |
| 12. | Cold Bokkeweld, Cape of Good Hope, - - | Oct. 13, 1838. |
| 13. | Little Piney, Gasconade county, Missouri, U. S. | Feb. 13, 1839. |
| 14. | Chateau Renard, Dept. du Loire, France, - | June 12, 1841. |

*Appendix to Meteoric Stones.*

| | | |
|---|---|---|
| 15. | Iwan, Hungary, - - - - - - | Aug. —, 1841. |

*Meteoric Irons.*

| | | Date of discovery. |
|---|---|---|
| 16. | Krasnojarsk, Siberia, - - - - - - - - - | 1772. |
| 17. | Xiquipilco, Toluca, Mexico, - - - - - - - | 1784. |
| 18. | Bitburg, in the Eifel, - - - - - - - - - | 1805. |
| 19. | Lenarto, Hungary, - - - - - - - - - | 1814. |
| 20. | Texas, (Red River), - - - - - - - - - | 1814. |
| 21. | Lockport, New York, U. S. - - - - - - - | 1818. |
| 22. | Burlington, Otsego county, New York, U. S. - - | 1819. |
| 23. | Guilford, North Carolina, U. S. - - - - - - | 1820. |
| 24. | Atacama, Bolivia, S. A. - - - - - - - - | 1827. |
| 25. | Bohumilitz, Bohemia, - - - - - - - - - | 1829. |
| 26. | Claiborne, Alabama, U. S. - - - - - - - - | 1838. |
| 27. | Cocke county, Tennessee, U. S. - - - - - - | 1839. |
| 28. | Asheville, Buncombe county, North Carolina, U. S. - | 1839. |
| 29. | Green county, Tennessee, U. S. - - - - - - | 1842. |
| 30. | St. Augustine's Bay, Madagascar, - - - - - - | 1843. |
| 31. | Arva, Hungary, - - - - - - - - - - | 1843. |

*Appendix to the Meteoric Irons.*

| | | |
|---|---|---|
| 42. | Scriba, near Oswego, New York, U. S. - - - - - | 1830. |

"P. S. Mr. Shepard offers specimens of Nos. 20, 21, 22, and 32, in exchange for any localities not included in this catalogue."

